# Race or racial segregation? Modification of the PM_2.5_ and cardiovascular mortality association

**DOI:** 10.1371/journal.pone.0236479

**Published:** 2020-07-27

**Authors:** Maayan Yitshak-Sade, Kevin J. Lane, M. Patricia Fabian, Itai Kloog, Jaime E. Hart, Brigette Davis, Kelvin C. Fong, Joel D. Schwartz, Francine Laden, Antonella Zanobetti

**Affiliations:** 1 Exposure, Epidemiology, and Risk Program, Department of Environmental Health, Harvard T.H. Chan School of Public Health, Boston, MA, United States of America; 2 Department of Environmental Health, Boston University School of Public Health, Boston, MA, United States of America; 3 Department of Geography and Environmental Development, Faculty of Humanities and Social Sciences, Ben-Gurion University, Beer-Sheva, Israel; 4 Channing Division of Network Medicine, Department of Medicine, Brigham and Women’s Hospital and Harvard Medical School, Boston, MA, United States of America; 5 Department of Social and Behavioral Sciences, Harvard T.H. Chan School of Public Health, Boston, MA, United States of America; 6 School of Forestry & Environmental Sciences, Yale University, New Haven, CT, United States of America; 7 Department of Epidemiology, Harvard T.H. Chan School of Public Health, Boston, MA, United States of America; Universidad del Desarrollo, CHILE

## Abstract

**Background:**

Many studies have identified an inequitable distribution of exposure to PM_2.5_ (particulate matter less than 2.5 microns) by race. We investigated the association of PM_2.5_ and cardiovascular mortality considering both the decedents’ race and neighborhood racial composition as potential modifiers.

**Methods:**

We obtained geocoded cardiovascular mortality records of all black and white decedents from urban block-groups in Massachusetts between 2001 and 2011 (n = 130,863). We examined the association between PM_2.5_ and cardiovascular mortality, and assessed effect modification by three types of racial modifiers: decedents’ race, census block-group percent black and white, and two novel measures of racial segregation. The Racial Residential Segregation (RRS) quantifies the concentration of non-Hispanic blacks and whites in each block-group. The Index of Racial Dissimilarity measures dissimilarity in non-Hispanic black and white racial distribution between the smaller census block-group and larger tract.

**Results:**

We found a 2.35%(95%CI: 0.92%;3.79%) increase in mortality for each 10μg/m^3^ increase in two-day average exposure to PM_2.5_. The effect was modified by the block-group racial composition, with higher risks in block-groups with the highest percentage of black residents (interaction p-value = 0.04), and in block-groups with the lowest RRS (i.e. higher black to white resident ratio, interaction p-value = 0.072). Racial dissimilarity did not modify the associations.

**Conclusion:**

Current levels of PM_2.5_ are associated with increased cardiovascular deaths in Massachusetts, with different risks between areas with different racial composition and segregation. This suggests that pollution reductions in neighborhoods with the highest percentage of non-Hispanic blacks would be most beneficial in reducing cardiovascular mortality and disparities.

## Introduction

Despite its declining trend in in earlier years, cardiovascular disease (CVD) mortality is currently the most common cause of death in the United States [1, 2]. Substantial disparities in CVD incidence and mortality persist among racial and ethnic minorities, particularly black Americans [3, 4], and while disparities in known lifestyle factors such as diet, exercise, and smoking account for a portion of this disparity, adjusting for individual behaviors does not fully account for these differences [5].

Research examining racial/ethnic CVD inequities suggests that structural differences, such as differential exposure to physical and environmental hazards, and increased susceptibility to disease may account for these unexplained associations [6]. Epidemiologic research on racial segregation—defined as the physical separation of households by race in space—shows that segregation persists in the United States, with white residents often living in predominately white neighborhoods, and black residents living in primarily black neighborhoods. Research on health patterns among those who live in segregated neighborhoods suggest worse physical and mental health outcomes for all residents in largely non-white neighborhoods—indicating contextual, not simply compositional effects of living in segregated areas [6, 7]. The structural mechanism that places non-white residents at particular risk includes intentional local and federal policies which have led to non-white communities being in closer proximity to highways, Environmental Protection Agency (EPA) Superfund sites [8, 9], and to be zoned for non-residential use [10]. In addition to greater physical exposures, these communities are characterized by fewer opportunities for social mobility, and fewer resources that can mitigate harms such as financial security, adequate healthcare, and sustained employment [6, 11]. Racial/ethnic minorities, particularly blacks, also experience a greater number of psychosocial stressors, with greater severity or duration than their white counterparts. As chronic stress is also known to weaken the immune function and accelerate cellular aging, the general susceptibility hypothesis would suggest even greater harm following health-harming exposures among non-white residents in segregated communities [12, 13].

Of particular interest for this study is ambient particulate matter smaller than 2.5 μm in diameter (PM_2.5_). The relative size and varied chemical composition of PM_2.5_ and finer air pollutants make both respiratory and cardiovascular toxicity likely through inhalation and even passage into the blood stream [14]. While the elderly and those with existing respiratory and cardiovascular conditions may be most susceptible to the cardiovascular effects of PM_2.5_, even among healthy individuals PM_2.5_ has been shown to impact heart rate, blood pressure, and vascular tone. Among those with comorbidities, PM_2.5_ has been associated with the progression of atherosclerosis [14]. As such, PM_2.5_ is a known causal risk factor for CVD morbidity and mortality experienced on the environmental level [15–20]. Studies suggest that non-whites are more likely to be exposed to higher levels of ambient PM_2.5_ than their non-white counterparts [21–25], while both race and local racial environment modify the relationship of PM_2.5_ exposure and mortality [26–29]. However, to our knowledge, no study so far has investigated the modification of the effect of PM_2.5_ exposure on CVD mortality comparing different ways to characterize race and racial environment.

Our objectives were to assess the association between short-term exposures to PM_2.5_ and CVD mortality and investigate the potential for effect modification by three racial modifiers: the decedents’ race, the census block group racial composition, and two novel racial segregation metrics.

The advantage of testing individual race as a modifier is in its specificity for the subject. This measure, however, do not consider a person’s surrounding local racial environment. Census block group racial composition does consider the racial environment; however, these measures define characteristics of the neighborhood independent of how the individual may interact with them. The degree to which each person’s individual race differs from the majority of the neighborhood in which they live may influence their health. Therefore, racial segregation metrics may better reflect the individual psychosocial experience of neighborhood stressors than race or racial composition. The racial residential segregation (RRS) measure tested in our study reflects the individual psychosocial experience of racial disparities as a result of segregation within the block group. The index of racial dissimilarity (IRD) reflects the individual psychosocial experience of racial disparities as a result of racial segregation of their block group compared to its surrounding neighborhoods.

## Methods

### Study population

We obtained CVD mortality records for all decedents 40 years and older who reside in urban block groups from the Massachusetts Department of Public Health for the years 2001–2011. The data used for this analysis includes confidential information, and identifying information, and therefore cannot be shared. These records provided information on residential address, place of death and sociodemographic information. We included all CVD mortality cases (ICD 10 group category I). We excluded rural areas due to insufficient variability in exposure and racial composition of the block groups.

This study was approved by the Harvard T.H. Chan School of Public Health Human Subjects Committee and by the Massachusetts State Department of Public Health.

### Environmental data

Mean daily PM_2.5_ concentrations from 2001–2011 were estimated for each 1x1 km grid cell covering Massachusetts by calibrating Aerosol Optical Depth (AOD) with monitor PM_2.5_ data, using mixed effect models (mean out-of-sample “ten-fold” cross-validation R^2^  =  0.88) [30]. Similarly average daily temperature was estimated from models incorporating daily moderate resolution imaging spectroradiometer (MODIS) land surface temperature data and land use regression variables (mean out-of-sample “ten-fold” cross-validation R^2^  =  0.94) [31]. Exposure predictions for each decedent were assigned from the grid cell closest to the geocoded residential address.

### Racial modifiers

We defined three types of potential racial modifiers: (1) the decedents’ race (black or white), (2) neighborhood racial composition (proportion of black and white residents from the US census, obtained at the block group level from the 5-year 2014 American Community Survey), and (3) neighborhood measures of racial segregation and dissimilarity (explained below). We defined the neighborhood as the block group of residence. This allows us to analyze the finest neighborhood spatial resolution using census measures of racial composition.

Neighborhood racial segregation and dissimilarity measures were developed from formulas published by Krieger et al. [32], and include the racial residential segregation (RRS) and the index of racial dissimilarity (IRD). We computed the dissimilarity index as suggested by Krieger et al [32–34], and generated a modified version of the RRS score across Massachusetts at the block group scale. S1 Table provides a short description of each index. The RRS ranges between -1 and 1 and provides a measure of the concentration of two races within the block group. Lower RRS (~ -1) indicates higher black to white resident ratio and higher RRS (~ 1) indicates lower black to white resident ratio The IRD ranges between 0% and 100% and compares the distributions of two races (non-Hispanic black and white) in a smaller area (e.g. block groups) to the distribution in a larger area (e.g. census tracts). Higher IRD indicates higher dissimilarity in the distribution of black residents between the census block group and tract.

While our study time-frame ranges from 2001–2011 we used the 2010 census block groups and tracts throughout all years of our analysis to maintain spatial consistency. Block groups contain between 600 and 3,000 people and are clustered within a single tract, while a tract contains between 1,200 and 8,000 residents. Both spatial scales of census data are considered to be fairly homogenous along socio-demographic characteristics and living conditions [35].

We expect all the racial modifiers to follow a consistent direction. However, each group tests a slightly different hypothesis. The first, decedents’ race, tests whether decedents of black race are more vulnerable to the PM_2.5_ effect. The second, whether decedents living in neighborhoods with higher percentages of blacks or whites were more vulnerable to the PM_2.5_-CVD mortality effect. The last tests whether living in neighborhoods of higher racial residential segregation or dissimilarity impacts the PM_2.5_ effect on CVD mortality.

### Statistical analysis

We assessed the association between CVD mortality and PM_2.5_ using the time-stratified case-crossover approach [36], a design for analyzing acute health effects of time-varying exposures in which each case serves as their own control by comparing their exposure of the event day with exposure on matched control days. Instead of analyzing why the outcome has occurred in one person compared to the other, this method investigates why the event happened in this day compared to other days, depending on the exposure. We defined the case day as the date of death, and selected control days as every 3^rd^ day before and after the case day, within the same month and year. Because the event day and the matched control days are within the same month and year, this approach controls for long-term temporal confounders and individual confounders by design. Since most studies found the strongest short term effects with mean PM_2.5_ in the current and previous day [37], we defined this as the exposure window for the main analysis. We tested the associations with exposure on the date of death and up to two preceding days as a sensitivity analysis. We conducted a second sensitivity analysis to test the association with PM_2.5_ in models restricted to periods where PM_2.5_ was below the current National Ambient Air Quality Standard (35 μg/m^3^ 24 hour average). We utilized conditional logistic regressions to assess the association between PM_2.5_ and mortality and adjusted all models for temporal confounders that were not controlled for by design (day of the week, linear and quadratic terms of temperature with the same exposure window as PM_2.5_).

We assessed modification of the PM_2.5_ and mortality association by adding multiplicative interaction terms with each of the racial modifiers. We assessed the association with each interaction term separately. We present the results as percent increase in CVD mortality risk and 95% CI for each 10μg/m^3^ increase in PM_2.5_. For the neighborhood racial percentages and the IRD, given that these are continuous variables, we present the results for a 10μg/m^3^ increase in PM_2.5_ at the 10^th^, 50^th^ and 90^th^ percentiles of the modifier, to show the trend in the exposure-response relationship. We categorized the RRS into 5 groups: the first two groups indicate predominantly non-Hispanic black residents in the block group (-1 to -0.5 and -0.5 to -0.1), the middle group (-0.1 to 0.1) indicates equal distribution of non-Hispanic white and black populations, and the last two groups (0.1 to 0.5 and 0.5 to 1) indicate predominantly non-Hispanic white residents in the block group. We performed the analyses using the clogit package in R version 3.2.4 [38].

## Results

We included 130,863 decedents in our analyses; 96.1% were white, 46.9% were males, 44.4% died at the hospital, 15.2% had a college education or more and the mean age at death was 80.2 years. The white population was older, with higher proportion of decedents with college education or more (Table 1). The population included in our study resided in 4,613 block groups in Massachusetts. The average of CVD mortality cases per block group was 288 cases, with a minimal number of 9 cases and a maximal number of 3,162 cases per block group.

**Table 1:** Population characteristics of decedents from urban block groups in Massachusetts from 2001–2011.

Population characteristics	All (N = 130,863)	White	Black
		N = 125,759 (96.1%)	N = 5,104 (3.9%)
**Male gender, n (%)**	61,355 (46.9)	58,786 (46.7)	2,569 (50.3)
**Education**			
*Elementary*	24,847 (19.2)	23,707 (19.1)	1,140 (22.9)
*High school*	69,321 (53.7)	66,533 (53.6)	2,788 (55.9)
*some college*	15,327 (11.9)	14,780 (11.9)	547 (11.0)
*College or more*	19,654 (15.2)	19,140 (15.4)	514 (10.3)
**Place of death, n (%)**			
*Hospital*	58,085 (44.4)	55,737 (44.0)	2,712 (53.1)
*Out of the hospital*	72,725 (55.6)	70,335 (55.9)	2,390 (46.8)
*Unknown*	53 (0.0)	51 (0)	2 (0)
**Age, Mean (SD)**	80.2 (12.5)	80.5 (12.3)	72.3 (14.5)

We present the summary statistics of the modifiers by PM_2.5_ in Table 2. The percent of black population of each census block group was higher in the block groups with higher PM_2.5_ concentrations. The median and IQR of RRS was near 1, indicating the majority of urban block groups in Massachusetts were majority white non-Hispanics. The IRD had a median number of 14.6% indicating low dissimilarity in the white/black racial distributions between the census block groups and tracts. Both RRS and IRD values were similar across the PM_2.5_ quartiles (Table 2).

**Table 2:** Summary statistics of race modifiers by block groups, divided by PM_2.5_ quartiles.

	PM_2.5_ quartiles across all block groups				
Modifier	All	Q1	Q2	Q3	Q4
**Individual Race, n (%)**					
*White*	125,759 (96.1)	30,699 (94.0)	31,410 (92.4)	32,017 (91.9)	31,633 (91.9)
*Black*	5,104 (3.9)	1,986 (6.0)	2,586 (7.6)	2,838 (8.1)	2,798 (8.1)
**Census race distribution, Median (IQR)**					
Percent white (%)	88 (20)	90 (17)	88 (20)	88 (21)	87 (22)
Percent black (%)	1.7 (6.2)	1.4 (5.2)	1.8 (6.4)	1.9 (6.5)	1.9 (7.0)
**Racial segregation and dissimilarity, median (IQR)**					
RRS	0.8 (0.2)	0.9 (0.2)	0.8 (0.3)	0.8 (0.3)	0.8 (0.3)
IRD (%)	14.6 (10.0)	14.9 (10.0)	14.6 (9.9)	14.5 (9.9)	14.5 (9.9)

IQR = Interquartile range; RRS = Racial residential segregation, range -1 to 1; IRD = Index of racial dissimilarity, range 0 to 100%.

We observed an increase in CVD mortality associated with the two-day average exposure to PM_2.5_ (2.35% increase, 95% CI 0.92%; 3.79%, per 10 μg/m^3^ increment in exposure). In sensitivity analyses, we also found associations with PM_2.5_ on the current (1.36% increase, 95% CI 0.18%; 2.55%) and previous day (1.84% increase, 95% CI 0.79%; 3.17). When we restricted the analysis to periods where PM_2.5_ was below the current EPA 24 hour standard (35 μg/m^3^) we found a similar association (2.60% increase, 95% CI 1.11%; 4.11%).

### Modification effects

#### Decedent race

We observed a higher percent change of CVD mortality among black decedents compared to whites (4.78% increase, 95% CI -1.99%; 12.02% vs. 2.25% increase, 95% CI 0.80%; 3.23), even though the decedents race was not a statistically significant modifier (p = 0.48).

#### Census-level racial composition

**Fig 1:**
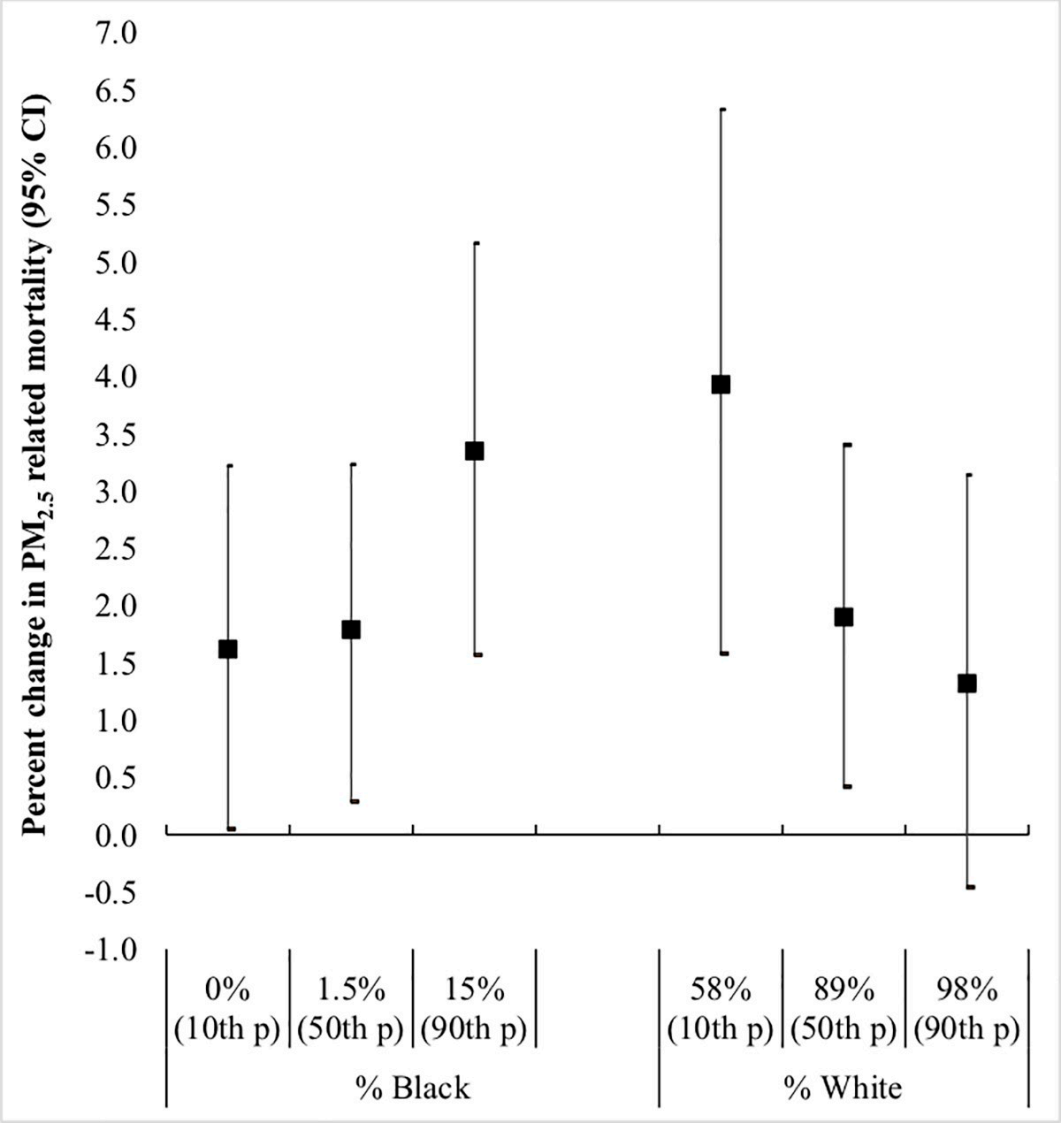
Percent change in CVD mortality risk per 10μg/m^3^ of PM_2.5_ among decedents in Massachusetts (2001–2011): Modification by census block group racial composition. The p values for the interactions between PM_2.5_ and percent black or white residents in the census block group were p = 0.04 and p = 0.07 respectively.

The strength of the associations with PM_2.5_ across the range of the census race distributions was of a consistent direction, with stronger associations in neighborhoods with higher proportion of black residents (interaction p value = 0.04) and lower percent of white residents (interaction p value = 0.07). For example, an increase of 10 μg/m^3^ of PM_2.5_ was associated with a 1.62% increase in CVD mortality (95% CI 0.05%; 3.22%) at the 10^th^ percentile of block group percent black (0%) and with a 3.35% increase in CVD mortality (95% CI 1.57%; 5.16%) at the 90^th^ percentile of block group percent black (16%) (Fig 1).

#### Racial segregation and dissimilarity

**Fig 2:**
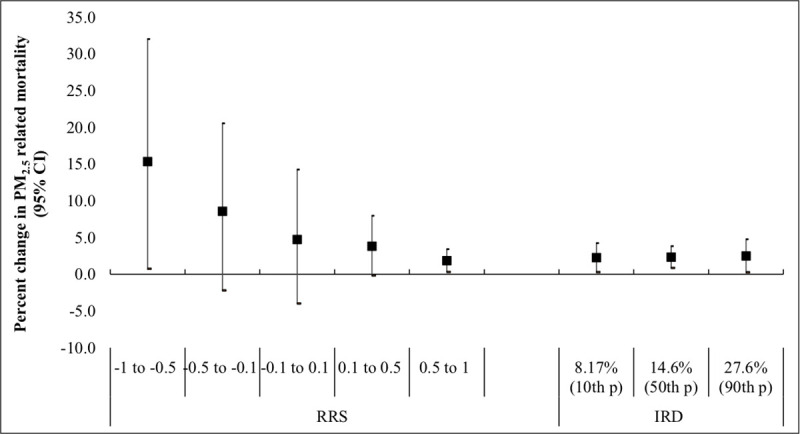
Percent change in CVD mortality risk per 10μg/m^3^ of PM_2.5_ among decedents in Massachusetts (2001–2011): Modification by Racial Residential Segregation (RRS) and Index of Racial Dissimilarity (IRD). For RRS, the first two groups indicates predominantly black non-Hispanic residents in block group (-1 to -0.5 and -0.5 to -0.1), the middle group (-0.1 to 0.1) indicates equal distribution of white and black non-Hispanic populations, and the last two group (0.1 to 0.5 and 0.5 to 1) indicate predominantly white non-Hispanic residents in the block group. The first group was considered as a reference group. The IRD ranges between 0% and 100% and compares the distributions of race in the block groups and the census-tracts. Higher IRD indicates higher dissimilarity in the distribution of blacks between the block group and the census tract. The p values for the interactions between PM_2.5_ and RRS were p = 0.48 for RRS -0.5 to -0.1, p = 0.23 for RRS -0.1 to 0.1, p = 0.142 for RRS 0.1 to 0.5, and p = 0.072 for RRS 0.5 to 1. The p value for the interaction between PM_2.5_ and IRD was p = 0.811.

PM_2.5_ –related risks of CVD death were lower for decedents living in neighborhoods with predominantly white residents. In the most black-segregated group (RRS 0.5 to 1), each 10μg/m^3^ increase in PM_2.5_ was associated with 1.84% increase in CVD mortality (95% CI 0.31%; 3.40%). In the least black-segregated group (RRS -1 to -0.5), each 10ug/m^3^ increase in PM_2.5_ was associated with 15.37% increase in CVD mortality (95% CI 0.76%; 31.99%, interaction p value = 0.072). IRD did not modify the association (interaction p value = 0.881) (Fig 2).

## Discussion

In this study, we observed an increased risk for CVD mortality associated with exposure to PM_2.5_. These risks were statistically significant and persisted when we restricted our analysis to days below the current 24-hour ambient air quality standards. Mortality risks were greater in block groups with higher percent of black residents, and in neighborhoods with higher black-segregation (i.e. higher black to white resident ratio). Dissimilarity in the distribution of blacks between the block group and the census tract did not modify the association.

There are several mechanisms through which racial disparities observed in this study may have come to exist. One key pathway is the disproportionate exposure to PM_2.5_. In this study, we found that black residents were more likely to live in areas at the highest PM_2.5_ quartile, and that as proportion of black residents in the census blocks increased, so did the relative ambient PM_2.5_. This is consistent with environmental justice literature which has found non-white communities are disproportionally exposed to pollution and polluting entities (i.e. factories, highways, etc.) compared to white communities. For instance, Kioumourtzoglou et al. examined the PM_2.5_-mortality association by city characteristics in 207 U.S cities and found higher risk in cities with higher percent of black residents [27]. Another U.S study that examined health disparities attributable to air pollution in Detroit, Michigan found that the burden of PM_2.5_ and other pollutants was disproportionate among non-white and low-income populations due to higher amount of industrial and traffic emissions [39]. Similarly, Fecht et al. found that neighborhoods in England and the Netherlands with more than 20% nonwhite residents had a statistically significant higher mean PM_10_ concentration [40]. Although the racial structure in England is not comparable to the U.S. this study strengths the findings of the U.S. studies.

Unlike similar analyses, in our study decedent race did not significantly modify the association between air pollutants and CVD mortality, even though race was a risk factor for CVD death. Wang et al found higher PM_2.5_ associated mortality rates among black and other races, compared to white race [28]. Similarly, Di et al assessed the effect of PM_2.5_ on mortality among older adults and found a statistically significant higher risk among nonwhite individuals compared to white (1.27% and 1.01% percent increase of daily mortality per 10-μg/m3 increase in PM_2.5_) [26]. It is likely that we were underpowered to measure this modification due to the relatively low proportion of black decedents, as well as the even lower proportion of black decedents in predominantly white census blocks. It is possible that systematically lower socioeconomic status, fewer access to salutary resources, and disproportionate exposure to psychosocial stressors would also explain the racial disparities in pollution-related CVD mortality found in this analysis [3, 13]. Racial differences in access to and participation in physical activity, and health-supporting nutrition, might also account for these differences [12, 21, 41, 42].

What makes our study particularly novel is the measures of residential segregation, beyond racial composition, in the examination of ambient air pollution and CVD mortality. Since 1970, racial segregation in the U.S has decreased modestly, although several major cities have experienced similar or increasing residential segregation [13]. While racial composition at a chosen geographic level is used most often (i.e. percent black in the census tract), this measure provides little information about the distribution of people in space or the historic processes which created the composition—which is a particularly important component of the creation and maintenance of segregation seen today. Racial composition alone also treats the sub-geographic patterns as independent of the larger geographic area, which cannot adequately capture the experience of isolation or exclusion which also characterizes racial segregation. As such, in this study we chose to examine other measures of residential segregation in addition the composition of the census block, specifically those which incorporate the degree to which racial groups are evenly distributed in space [13, 34]. We used two of these measures (the RRS and the IRD) to assess the modification of the PM_2.5_ effect on CVD mortality by racial segregation and dissimilarity and found lower PM-mortality risks in neighborhoods with many non-Hispanic whites and few black residents. This can be attributed to the lower exposure level and the better access to health care and resources that is more common in predominantly white neighborhoods [21]. We did not find IRD to modify the PM-mortality risks, suggesting that in this Massachusetts population the PM_2.5_ effect does not change when taking the residential patterning of the larger geographical area into account.

A recent systematic review found that in most studies, the health effects associated with segregation (measured using the index of racial dissimilarity between a smaller and larger geographic area) among the black population were not detected among whites in the same cities [13]. Moreover, evidence from studies that assessed the effect of decreasing segregation on health suggests black would benefit more from decreasing segregation than do whites [43–45]. Unfortunately, we did not have sufficient statistical power to explore the differences in the modification effects of racial segregation, by the individual race.

The greatest strength of this study is the assessment of the modification of the PM_2.5_ –CVD mortality risk by race and residential segregation. This study incorporates an analysis of three different racial modifiers–race, the commonly used racial neighborhood composition, and novel measures of racial segregation and dissimilarity. Our study had several limitations. First, the choice of the geographic scale to represent the person’s environment is a key methodological issue that arises when studying the neighborhood effects on health [46]. The geographical boundaries defined in the study often do not capture the boundaries that are meaningful to the residents [5, 46] and may cause exposure misclassification. Second, similar to other studies that assess the association with air pollution; there is a possibility of exposure measurement error. However, due to our use of highly spatially and temporally resolved models to estimate individual exposure to PM at the residential address, we expect the error to be minor and non-differential. Finally, because the majority of the population is Massachusetts are non-Hispanic white, the analysis of modification by individual race was underpowered.

In conclusion, current levels of PM_2.5_ are associated with increased cardiovascular deaths in Massachusetts, with larger risks across different indicators of non-Hispanic white and black racial composition and segregation. This suggests that pollution reductions in neighborhoods with highest percentage of non-Hispanic blacks and highest segregation would be most beneficial in reducing cardiovascular mortality and related disparities.

## Supporting information

S1 Table Neighborhood racial segregation and dissimilarity measures. (DOCX)
