# Supplementary material for: Race or racial segregation? Modification of the PM2.5 and cardiovascular mortality association
Source: PLoS One. 2020 Jul 27;15(7):e0236479. doi: 10.1371/journal.pone.0236479 (PMC7384646; doi:10.1371/journal.pone.0236479)
Supplement: S1 Table — (DOCX) [file pone.0236479.s001.docx]

S1 Table. Neighborhood Racial Segregation and Dissimilarity Measures

| *Measure* | *Formula* | *Possible values* | *Interpretation* |
| --- | --- | --- | --- |
| Racial Residential Segregation (RRS) | RRS = (p_white_ - p_black_\|)/ p_total_  P_black_ =the number of the non-Hispanic blacks in the census block group.  p_white_ =the number of the non-Hispanic whites in the block group.  p_total_ =the total of non-Hispanic blacks and whites in the block group. | Range from -1 to 1. | Higher values indicate predominantly non-Hispanic white residents in the block group, negative values indicates predominantly non-Hispanic black residents in block group, and zero indicates equal distribution of non-Hispanic whites and blacks. |
| Index of Racial Dissimilarity (IRD) | IRD = (0.5 Σ \|( p_black_ / P_black_) − p_white_ / P_white_)\|) * 100  P_black_ =the number of non-Hispanic blacks in the census block group.  P_black_ =the number of non-Hispanic blacks in the census tract.  p_white_ and P_white_ = corresponding values for the non-Hispanic whites in the block group and census tract. | Range from 0% to 100%, | High values indicate high dissimilarity in the distribution of non-Hispanic blacks to whites between the block group and the census tract, and low values indicate similarity. |
